# Characterization of a Stemness-Optimized Purification Method for Human Dental-Pulp Stem Cells: An Approach to Standardization

**DOI:** 10.3390/cells11203204

**Published:** 2022-10-12

**Authors:** Martin Philipp Dieterle, Tara Gross, Thorsten Steinberg, Pascal Tomakidi, Kathrin Becker, Kirstin Vach, Katrin Kremer, Susanne Proksch

**Affiliations:** 1 Division of Oral Biotechnology, Center for Dental Medicine, Medical Center—University of Freiburg, Faculty of Medicine, Albert-Ludwigs-University of Freiburg, Hugstetter Str. 55, 79106 Freiburg, Germany; 2 Department of Operative Dentistry and Periodontology, Centre for Dental Medicine Medical Center—University of Freiburg, Faculty of Medicine, Albert-Ludwigs-University of Freiburg, 79106 Freiburg, Germany; 3 G.E.R.N. Center for Tissue Replacement, Regeneration & Neogenesis, Medical Center—University of Freiburg, Faculty of Medicine, Albert-Ludwigs-University of Freiburg, 79108 Freiburg, Germany; 4 Institute of Medical Biometry and Statistics, Medical Center—University of Freiburg, Faculty of Medicine, Albert-Ludwigs-University of Freiburg, 79104 Freiburg, Germany; 5 Department of Oral and Maxillofacial Surgery, Center for Dental Medicine, Medical Center—University of Freiburg, Faculty of Medicine, Albert-Ludwigs-University of Freiburg, 79106 Freiburg, Germany

**Keywords:** stem cells (MeSH ID D013234), dental pulp (MeSH ID D003782), cell separation (MeSH ID D002469), stem cell research (MeSH ID D057905), primary cell culture (MeSH ID D061251), dentistry (MeSH ID D003813), tissue engineering (MeSH ID D023822)

## Abstract

Human dental pulp stem cells (hDPSCs) are promising for oral/craniofacial regeneration, but their purification and characterization is not yet standardized. hDPSCs from three donors were purified by magnetic activated cell sorting (MACS)-assisted STRO-1-positive cell enrichment (+), colony derivation (c), or a combination of both (c/+). Immunophenotype, clonogenicity, stemness marker expression, senescence, and proliferation were analyzed. Multilineage differentiation was assessed by qPCR, immunohistochemistry, and extracellular matrix mineralization. To confirm the credibility of the results, repeated measures analysis and post hoc *p*-value adjustment were applied. All hDPSC fractions expressed STRO-1 and were similar for several surface markers, while their clonogenicity and expression of CD10/44/105/146, and 166 varied with the purification method. (+) cells proliferated significantly faster than (c/+), while (c) showed the highest increase in metabolic activity. Colony formation was most efficient in (+) cells, which also exhibited the lowest cellular senescence. All hDPSCs produced mineralized extracellular matrix. Regarding osteogenic induction, (c/+) revealed a significant increase in mRNA expression of *COL5A1* and *COL6A1*, while osteogenic marker genes were detected at varying levels. (c/+) were the only population missing *BDNF* gene transcription increase during neurogenic induction. All hDPSCs were able to differentiate into chondrocytes. In summary, the three hDPSCs populations showed differences in phenotype, stemness, proliferation, and differentiation capacity. The data suggest that STRO-1-positive cell enrichment is the optimal choice for hDPSCs purification to maintain hDPSCs stemness. Furthermore, an (immuno) phenotypic characterization is the minimum requirement for quality control in hDPSCs studies.

## 1. Introduction

The dental pulp is located in the central pulp cavity of each tooth, called the pulp chamber, and contains a complex histological architecture. It comprises a heterogeneous cell population represented by fibroblasts, endothelial cells, perivascular cells, neural cells (e.g., Schwann cells and perineural cells), odonto-osteoprogenitors, inflammatory and immune cells as well as stem cells (SCs) [1,2,3,4]. The dental pulp functions by supplying the teeth with blood and nutrients, synthesizing dentin, reception and transmission of sensory cues, and cellular as well as humoral immunity. Diseases of the pulp, such as chronic or irreversible pulpitis, usually entail a complete pulp removal during endodontic treatment, which often results in tooth loss in the long run [5]. Thus, efficient therapeutic strategies to preserve and repair the dental pulp are urgently needed in clinical practice.

Tissue engineering of the dental pulp and neighboring oral tissues is challenging due to tooth anatomy and the limited blood supply from the root ends. This may result in nutrient deficits in coronal regions [6] and hinders the therapeutic success. Nonetheless, many cell- or biomaterial-based therapeutic approaches have been presented in the literature [7,8,9,10,11]. Exemplarily, Liu et al. recently described a dopamine-modified, hyaluronic-acid based biopolymer, which enables efficient adhesion of dental pulp stem cells (DPSCs) for dental pulp regeneration and dentin synthesis [12]. Albeit promising, major challenges in regenerative endodontics still need to be overcome [12]. Especially the rejuvenation of aged teeth/dental pulps, the optimal cell type for regeneration, and efficient cell isolation, purification, disinfection, and transplantation are still a matter of debate [13].

Ideally, cells that are administered for dental pulp regeneration purposes would support dentin synthesis, enable an efficient dentin-pulp/soft tissue interaction, withstand hypoxia, facilitate anti-inflammatory and regenerative molecular signaling pathways, while building a hard tissue barrier towards potentially inserted repair materials [14,15,16,17,18,19,20,21]. In the light of their proliferative capacity, their developmental potential, as well as their self-regenerative properties, SCs are optimal candidates for such applications. Of note, the dental pulps harbor their own SC population, called DPSCs (see above [22]). DPSCs were described for the first time by the pioneering work of Gronthos et al., who harvested pulp cells without any further purification based on surface marker expression [23]. Embryologically, DPSCs are most likely derivates from the neural crest, and thus are of ectodermal origin [24,25]. From a molecular point of view, this is an intriguing cell fraction since it unites surface marker expression of mesenchymal SCs (e.g., cluster of differentiation (CD) 10, CD44, CD90, CD146, CD166, STRO-1), neural SCs (NES, SOX2), and embryonic SCs (octamer binding transcription factor 4 [Oct-4], Nanog) [26,27,28,29,30]. Functionally, the developmental complexity of the dental pulp explains why it harbors very different cell populations. The latter also indirectly reflect the multilineage differentiation potential of DPSCs [31], i.e., they can differentiate into odontoblasts, osteoblasts, cementoblasts, chondrocytes, neural cells, myoblasts, endothelial cells, and adipocytes in vitro and in vivo [32,33,34,35,36,37,38,39,40,41]. The molecular mechanisms behind the respective lineage decisions are complex and yet incompletely understood. The response of DPSCs to stimuli such as fetal calf serum or hyaluronic acid fragments, which result from the degradation of biomaterial scaffolds for cell transplantation, also need further characterization [42,43]. For example, it was recently shown that hypoxia is an adequate cue to induce neural cell-like differentiation of DPSCs. Moreover, conditioned medium from hypoxia-treated DPSCs can induce neural differentiation of undifferentiated DPSCs in a paracrine manner [33]. Endothelial differentiation and stemness maintenance of DPSCs is, among others, regulated by a p53-p21-Bmi-1 signaling axis [41]. Moreover, DPSCs are thought to function as pericytes/pericyte-like cells and thus their interaction with endothelial cells is of great importance for angiogenesis as well [44]. These features are well known from MSCs and might also apply to DPSCs. The latter have been shown to produce vascular endothelial growth factor (VEGFA) [45], other growth factors such as basic fibroblast growth factor (FGFR1) [46], matrix remodeling enzymes that may facilitate endothelial cell migration [47], and to support pericyte recruitment [48] as well as the stabilization of newly formed vessels [49,50,51]. Hence DPSCs are widely accepted as the best candidates for dental pulp regeneration [22]. However, no specific DPSC surface marker has been established so far, which makes their isolation and characterization laborious and error prone.

Technically, the first step to address dental pulp regeneration is the isolation of a defined and sufficient cell fraction from a pulp chamber. A multitude of scientific papers reported on DPSCs-like cells that were derived by different isolation methods with and without further purification steps. As a consequence, a huge amount of data has been raised and a bunch of characteristics were attributed to DPSCs, although the cell fractions used for different studies were quite heterogeneous, some remaining without any purification from the dental pulp cell mixture at all. DPSCs were found to be clonogenic, i.e., form colonies from single cells, and synthesize a mineralized matrix in vitro, and differentiate into odontoblasts in vivo [23]. Furthermore, they seem to have a low tumorigenic potential and exhibit a strong immuno-tolerance in vivo [52]. Interestingly, the same group that initially isolated DPSCs also reported that single-colony-derived dental pulp cell strains differed from each other regarding their odontogenic potential [53]. To achieve reproducible experimental setups, they introduced the DPSC enrichment based on the purification of STRO-1-positive (STRO-1+) cells, which is a widely used surface marker protein for mesenchymal/stromal SCs [54,55,56]. Apart from the enrichment based on the expression of signature molecules such as STRO-1 using magnetic or fluorescence-activated cell sorting (MACS or FACS, respectively), the limiting dilution technique can also be applied to purify DPSCs. It allows colonies to form from a single cell and enables further characterization of the colony-derived cells [57]. Although DPSCs ideally qualify for pulp regeneration, a standardized isolation protocol is missing so far. To the best of our knowledge, the most widely used purification methods, i.e., sorting vs. colony-formation, have never been directly compared in terms of DPSC properties and differentiation potential. This is, however, of vital importance for safe and efficient application of these SCs in the treatment of patients.

The lack of standardization in the purification of DPSCs has led to an extensive body of basic scientific literature, which is difficult to compare [58]. Consequently, very few clinical studies using DPSCs have been published so far. Nakashima et al. reported a study using mobilized autologous DPSCs for complete pulp regeneration based on preclinical laboratory studies on dogs [59,60]. Another preclinical study showed the usefulness of DPSCs in bone regeneration, which has been translated into a pilot clinical trial following the ethical approval [58]. Although there are first clinical trials using human DPSCs (hDPSCs) for pulp regeneration (NIH clinical trial registration numbers NCT04983225, NCT03386877, NCT02523651), a standardized protocol for the purification and enrichment of hDPSCs does not exist so far. This is noteworthy, because heading for the routine clinical application of DPSC-based therapies necessarily requires the compliance with standardized quality criteria [54,61].

**Figure 1:**
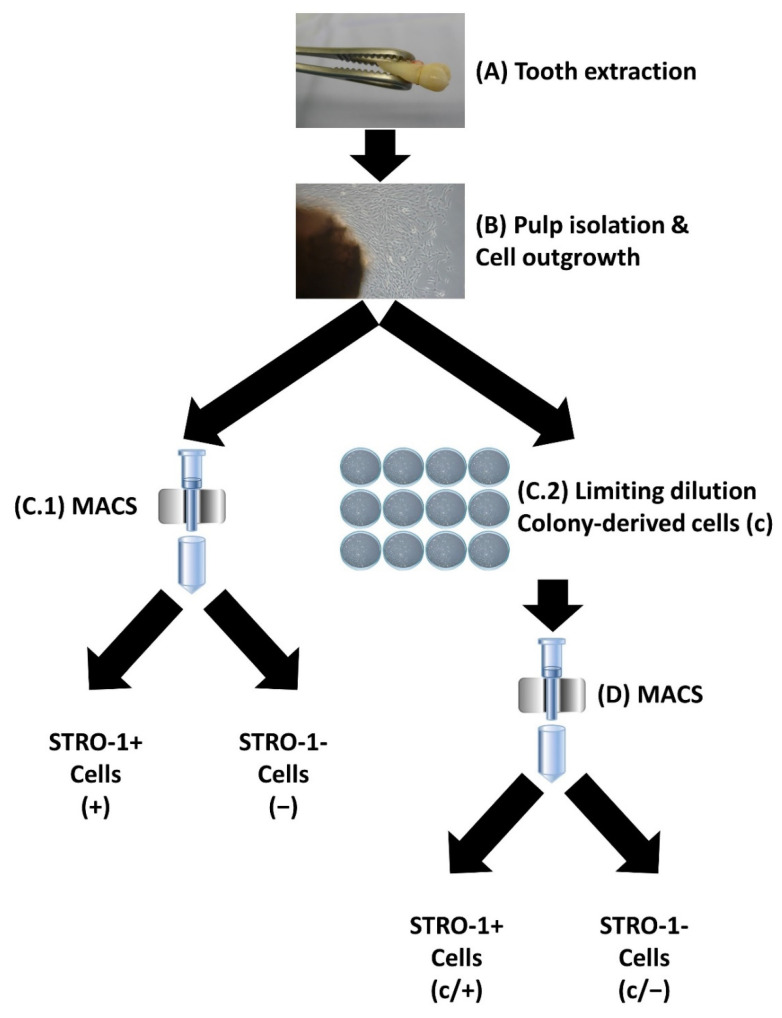
Schematic representation of the isolation and purification of human dental-pulp cells. (**A**) Teeth were extracted from healthy donors. (**B**) The dental pulp was isolated, and cells were allowed to grow out in a culture dish. (**C.1**) Following magnetic-activated cell sorting (MACS) with an anti-STRO-1 antibody, STRO-1 positive (STRO-1+) cells, designated as (+), and STRO-1 negative (STRO-1−) cells, called (−), were enriched. (**C.2**) Alternatively, cells were subjected to limiting dilution and colony-forming cell clones were isolated (c). (**D**) A subset of (c) cells was further purified via MACS, resulting in colony-forming STRO-1+ (c/+) and colony-derived STRO-1− (c/−) cells. Details are described in the Section 2 as well as in Section 3.1.

For this reason, it was the aim of the present study to perform a systematic comparison of three purification methods, focusing on the identification of a key stemness marker in donor-matched DPSCs, i.e., (i) the MACS-assisted enrichment of STRO-1-positive cells, (ii) the enrichment of colony-forming cells, and (iii) a combination of both methods (Figure 1). It was hypothesized that hDPSCs differ regarding the respective purification method in terms of standard criteria for SC properties, i.e., clonogenicity, surface marker expression, and multilineage potential [62,63,64]. Since clinical hDPSCs application requires a substantial number of cells, differently purified hDPSCs (in the following designated as DPSCs) were further characterized regarding their proliferation capacity and cellular senescence.

## 2. Materials and Methods

### 2.1. Cell Isolation

All experiments have been carried out in accordance with the guidelines of the World Medical Association Declaration of Helsinki and were approved by the Committee of Ethics of the Medical Faculty of the Albert-Ludwigs-University Freiburg, Freiburg, Germany (EK-153/15). Non-carious human premolar teeth and third molars were obtained from three healthy patients (two female [both 14 years old], one male [aged 20]) with written, informed consent. Pulp tissue was extracted under sterile conditions, touched with 10% iodide, thoroughly washed, and minced. Tissue fragments were plated as explants in MEMalpha medium (Life Technologies, Darmstadt, Germany) supplemented with 10% fetal bovine serum (Biochrom, Berlin, Germany), 1 % glutamax (Life Technologies), and 1% kanamycin (Sigma Aldrich, Munich, Germany). The medium was exchanged every 2–3 days until cell outgrowth. Cells were expanded by splitting (passaged (p) 2–4 times), and frozen in liquid nitrogen until usage.

### 2.2. Dental Pulp Stem Cell Purification

For the enrichment of STRO-1+ cells (+), dental-pulp cells were incubated with a mouse (ms) anti-human STRO-1 antibody (2 µg/10^6^ cells; R&D Systems, Minneapolis, MN, USA) diluted in cold rinsing buffer (phosphate-buffered saline (PBS), Life Technologies) with 2 mM EDTA (Sigma Aldrich) containing 0.5% bovine serum albumin (BSA; Sigma Aldrich) for 30 min at 4 °C. After washing, cells were incubated with anti-ms IgM microbeads (Miltenyi Biotec, Bergisch Gladbach, Germany) for 15 min at 4 °C. After a final washing step, (+) cells were brought into a magnetic field (MACs Multistand Miltenyi Biotec) using the MiniMACS™ separator (Miltenyi Biotec) containing paramagnetic beads. Subsequently, cells were removed from the magnetic field and collected in 1 mL buffer, using a plunger and kept in culture medium at 37 °C with 5% CO_2_.

For the retrieval of colony-derived cells (c), dental-pulp cells were plated in limiting dilution. Emerging colonies were photographed using a Leica DMIL inverted microscope (Leica Microsystems, Wetzlar, Germany) connected to a Leica D-Lux3 CCD camera (Leica Camera, Solms, Germany), and cells were subsequently collected.

For the enrichment of colony-derived STRO-1+ cells (c/+), dental-pulp cells were plated in limiting dilution, harvested from colonies, and subsequently enriched based on their STRO-1 expression, as described above.

### 2.3. Colony Forming Units (CFU) Assay

For CFU assays, cells were seeded in limiting dilution, i.e., 2 cells/cm^2^, incubated for 14 days, fixed with 4% formaldehyde, and stained with 0.1% Azur II (Sigma Aldrich, Taufkirchen, Germany) dissolved in Aq_dest_ for 20 min at room temperature (RT), air dried, and photographed with a SZH10 microscope (Olympus, Münster, Germany) equipped with a CCD Colour view III camera (Olympus, Münster, Germany). The resulting images were taken using the Cell Olympus cell Sens software version 1.5 (Olympus, Münster, Germany). Images were analyzed using the ImageJ plugin “analyze particles” following threshold adjustment. Stained colonies of ≥50 cell were scored as colony forming (CFU-F) and counted. CFU-F efficiency was calculated as follows:

CFU-F efficiency = (counted CFU-F/cells originally seeded) × 100

### 2.4. Metabolic Activity/Proliferation Assay

The metabolic activity and proliferation of the differently isolated DPSC fractions were measured using the resazurin/Alamar blue^®^ assay (Bio-Rad, Munich, Germany). Briefly, cells were seeded at 0.5 × 10^4^ cells/cm^2^ and at the indicated points in time (1, 3, 7, 14 and 21 days, respectively), culture medium was replaced by medium containing 10% (*w*/*v*) Alamar blue^®^ reagent. After incubation for 3 h at 37 °C and 5% CO_2_, triplicate samples of the supernatant were analyzed by measuring fluorescence according to manufacturer’s instructions (570 nm excitation and 630 nm emission wavelength) in an infinite-M microplate reader (Tecan, Männedorf, Switzerland). The relative amount of Alamar blue^®^ reduction in the samples was calculated using a 100% reduced Alamar blue^®^ control as a reference. Blanks and negative controls were routinely included in each run.

### 2.5. Flow Cytometry

For each run, a total of 1 × 10^6^ cells were incubated with a final concentration of 5 μg/mL each of fluorescein isothiocyanate- (FITC), phycoerythrin- (PE), peridinin chlorophyll protein complex (PerCP), or allophycocyanin (APC)-conjugated ms anti-human CD10, CD13, CD14, CD19, CD34, CD44, CD45, CD73, CD90, CD105, CD146, CD166, HLA-DR, c-kit, STRO-1, alkaline phosphatase antibodies (BD Biosciences, Heidelberg, Germany) or isotype-matched controls, respectively, (20 min, 4 °C in the dark). After intense washing and resuspension in PBS (Life Technologies), flow cytometric analysis was performed using a FACSCalibur (BD Biosciences) flow cytometer. Data were collected and analyzed with CellQuest software version 3.3 (BD Biosciences). For each run, 20,000 cell events were gated and fluorochrome spectral overlap was checked and compensated whenever required.

### 2.6. Senescence Assay

Cellular senescence was tested using a β-galactosidase staining kit (Cell Signaling, Frankfurt, Germany) according to manufacturer’s instructions. Briefly, cells were seeded at 1 × 10^5^/well in a 6-well-plate (Cellstar^®^, Greiner Bio-One GmbH, Frickenhausen, Germany) and cultivated to sub-confluence of approximately 70%. Cells were fixed, rinsed with PBS (Life Technologies), and incubated with β-galactosidase staining solution overnight at 37 °C in a dry incubator (FD-53, Binder GmbH, Tuttlingen, Germany). Plates were covered with 70% glycerol (Sigma Aldrich), photographed with an inverted microscope (Zeiss, Oberkocheln, Germany) connected to an Axiocam camera, and data were collected using the Zen Blue 2.3 software (Carl Zeiss Microscopy GmbH, Göttingen, Germany).

### 2.7. Multilineage Differentiation Assay

The following medium supplements were used in MEMalpha medium (Life Technologies; basic supplements, see above) to trigger the differentiation of isolated DPSCs into osteoblasts (50 μg/mL ascorbate-2-phosphate, 10 mM b-glycerophosphate, and 10^−7^ M dexamethasone, all chemicals from Sigma Aldrich) and chondrocytes [cell pellets cultivated with 1 ng/mL transforming growth factor-b (TGF-b; R&D Systems), 50 μg/mL ascorbate-2-phosphate, 1 × insulin transferring selenium supplement-x (Invitrogen, Darmstadt, Germany), and 10^−7^ M dexamethasone (Sigma Aldrich)] [65]. To induce neural differentiation of DPSCs, cells were cultured in growth medium supplemented with neural induction medium containing 2% B27, 2% N2 (PAA Laboratories, Coelbe, Germany), 25 ng/mL BDNF, 40 ng/mL NGF and 25 ng/mL bFGF (R&D Systems, Minneapolis, MN, USA) [66]. DPSC isolates were incubated in the respective differentiation medium for 21 d each. Next, polymerase chain reaction (RT-PCR) and immunofluorescence assays were employed to detect the respective differentiation into the desired lineage of DPSCs.

### 2.8. Indirect Immunofluorescence (IIF) and Immunohistochemical (IHC) Stains

Enrichment of STRO-1-expressing cells and cell differentiation were assessed by immunostaining using ms anti-human STRO-1 (1:100, R&D Systems), rabbit (rb) anti-human MAP2 (1:200, Abcam, Cambridge, UK), ms anti-human Col2a1 (1:50, Abcam), ms anti-human Aggrecan (1:100, Abcam), rb anti-human hTERT (1:50, Abcam), ms anti-human Nanog (1:50, Abcam), and rb anti-human Oct-4 (1:50, Abcam) antibodies, respectively. For IIF, unspecific binding sites were blocked with 5% BSA (Sigma Aldrich, St. Louis, MO, USA) and 0.2% (0.3% for Oct-4) Triton X-100 (Sigma Aldrich, St. Louis, Missouri, USA) in PBS buffer for 30 min at RT. Subsequently, the respective primary antibody was applied and detected by goat anti-mouse IgG1 Alexa Fluor 488 (1:200 in PBS) and 555 (1:40 in PBS) antibodies (Invitrogen) alternated by intense wash steps three times in PBS for 5 min each. For actin fiber staining, Texas Red-labelled phalloidin (Abcam, Cambridge, UK) was used and cell nuclei were counterstained using 4′,6-diamidin-2-phenylindole (DAPI, 1:1000 in PBS; Abcam, Cambridge, UK). After mounting (Fluoromount G, Southern Biotech, Birmingham, AL, USA), the cells were photographed with a Biozero BZ-9000 fluorescence microscope equipped with a CCD camera, and data were collected with the BZ II analyzer software (all Keyence Corp., Neu-Isenburg, Germany).

For IHC, samples were deparaffinized in descending alcohol series, and endogenous peroxidase was blocked by using 3% H_2_O_2_. Unspecific binding sites were blocked with 5 % BSA with 0.25% triton X-100 in normal horse serum for 60 min at RT. Sections were exposed to the anti-human Col2a1 antibody diluted in PBS-BSA 2% overnight at 4 °C, washed with PBS, and incubated with a biotinylated anti-mouse or anti-rabbit antibody, respectively, (Vector Laboratories, Burlingame, CA, USA). Subsequently, preformed avidin-biotin peroxidase complexes (ABC solution; Vectastain, Vector Laboratories) were added prior to antigen visualization by adding a freshly prepared peroxidase substrate solution containing 3,5-diaminobenzidine (Sigma-Aldrich, Munich, Germany). After a final wash step, slides were counterstained with hematoxylin, dehydrated and mounted with TechnoVit 7200 (Heraeus Kulzer, Wehrheim, Germany). Negative controls without primary antibodies were routinely included for each sample. The photographs were taken as described above.

### 2.9. Alizarin Red Staining and Extraction

Cells were fixed with ice-cold ethanol, washed with Aq_dest_, and incubated with 40 mM Alizarin red solution (Sigma Aldrich, pH 4.1) for 20 min at RT. After thorough washing, the air-dried specimens were evaluated with a SZH10 microscope (Olympus, Münster, Germany) equipped with a CCD Colour view III camera and the resulting images were taken and analyzed using the CellSens software version 1.5 (both Olympus, Münster, Germany). For quantification, Alizarin red was extracted with 10% acetic acid for 30 min at RT. The cell layer was scraped, and the solution was covered with mineral oil (Sigma Aldrich) and incubated for 10 min at 85 °C. The supernatant was transferred into microplates, the absorbance was read at 420 nm in triplicates, and data were collected and analyzed using the Magellan v6.2 software (Tecan, Crailsheim, Austria).

### 2.10. qPCR

Total cellular RNA was purified using a guanidium–thiocyanate method (RNeasy Mini kit; Qiagen, Hilden, Germany) and stored at −80 °C. The RNA integrity and quantity were checked using the Experion RNA StdSens chip microfluidic technology according to manufacturer’s instructions (Bio-Rad, Munich, Germany) and verified using the Nanodrop 1000 spectrophotometer (Thermo Scientific, Darmstadt, Germany). cDNA was synthesized from 80 ng total RNA for osteogenically induced cells and from 24 ng total RNA for neurogenically induced cells by using the RT^2^ PreAmp cDNA synthesis kit (Qiagen, Hilden, Germany), preceded by a genomic DNA elimination step at 42 °C for 5 min, in a C1000 Thermal Cycler (Bio-Rad). Subsequently, cDNA was synthesized at 42 °C for 30 min followed by 95 °C for 5 min. Pre-amplification was carried out using pre-validated RT^2^ Primer assays (Qiagen) at 0.12 µM in a 25 µL reaction volume at 95 °C for 10 min, followed by 8 cycles at 95 °C for 15 sec and 60 °C for 120 sec and a final incubation at 37 °C for 15 min and 95 °C a for 5 min after the addition of side reaction reducer. After dilution with nuclease-free H_2_O, cDNA was amplified in duplicates each using the same pre-validated RT^2^ Primer assays at 0.4 µM in a 25 µL reaction volume. Cycling conditions were as follows: 95 °C for 10 min followed by 40 cycles of 95 °C for 15 s and 60 °C for 60 s, using iQ SYBR Green mastermix in a CFX96 cycler (both Bio-Rad, Munich, Germany), according to manufacturer’s instructions. The products’ specificity of each amplicon was checked by examining the melting temperatures (heating at 0.05 °C/s to 95 °C). Negative reverse transcription and negative template controls were included in all PCR runs. Data were collected with CFX96 Manager Software version 1.0 (Bio-Rad), and relative quantities of the respective genes of interest were normalized to the relative quantity of *ACTB*, *RPL13A*, *RPLP0*, and *HPRT* as references, which were validated for Ct-value consistency (inclusion criteria: ΔCt < 0.5 irrespective of group and culture condition). Data were analyzed and plotted using the RT^2^ Profiler PCR array data analysis template, i.e., fold-change (2^(−^^ΔΔCt)^) described as fold-regulation, representing fold-change results in a biologically meaningful way (also see: https://www.qiagen.com/de/shop/genes-and-pathways/data-analysis-center-overview-page/; accessed on 18 May 2022; 14:22 GMT+1).

### 2.11. Statistics

For each condition and each time point, a linear mixed regression model was fitted to evaluate the influence for each outcome of interest (Alamar blue^®^ reduction rate, Alizarin red extraction, qPCR), and *t*-tests were used for pairwise comparisons. The resulting *p*-values were adjusted by applying the Bonferroni method to correct for multiple testing. Results were considered statistically significant if *p* < 0.05. The calculations were performed with the statistical software STATA 17.0 (StataCorp LLC., College Station, TX, USA).

## 3. Results

### 3.1. Magnetic-Activated Cell Sorting (MACS) and Colony Selection Give Rise to STRO-1+ Human Dental-Pulp Cells

The human dental pulp harbors different cell types including fibroblasts, odontoblasts, immune cells such as dendritic cells and macrophages, as well as stem cells (SCs), which are also known as human dental pulp stem cells (DPSCs) [67]. Generally, the various cell types can be distinguished by the expression of characteristic genes and the presence of surface marker proteins. Immunophenotyping of cells, i.e., characterizing them by methods such as fluorescence-activated cell sorting (FACS) or magnetic-activated cell sorting (MACS), is thus a common way to separate and purify different cell species. In the case of DPSCs, different purification methods have been presented in the literature, which, however, give rise to inconsistent cell populations with varying biological properties [68,69,70,71,72,73]. Consequently, a standardized isolation and purification protocol for DPSCs is urgently needed to allow for (i) the reliable and reproducible isolation of DPSCs, (ii) standardized reporting of DPSCs research, and (iii) the use of DPSCs in regenerative medicine. Thus, we compared different purification protocols, characterized the resulting human dental pulp cell populations, and compared them in the light of SC-defining properties.

Premolar and third molar teeth were extracted from healthy donors (Figure 1A) and the dental pulps were subsequently isolated (Figure 1B). Cell outgrowth was enabled in a culture dish (Figure 1B). MACS-dependent enrichment of STRO-1+ dental-pulp cells was performed as described in the Section 2 (Figure 1(C.1)). The resulting cell fraction is designated as (+) in the following; the corresponding eluate of STRO-1-negative control cells as (−). Alternatively, cells were subjected to limiting dilution (Figure 1(C.2)). After colony formation, cells were used for further experiments and are labeled as (c). A combination of both purification methods was applied to a third group of cells that initially underwent colony formation, followed by MACS using anti-STRO-1 antibodies (Figure 1D), giving rise to colony-forming STRO-1+ cells (c/+) and the corresponding control STRO-1− eluate, called (c/−). Thus, the different purification approaches gave rise to five different dental pulp cell groups, which were characterized further (Figure 1).

**Figure 2:**
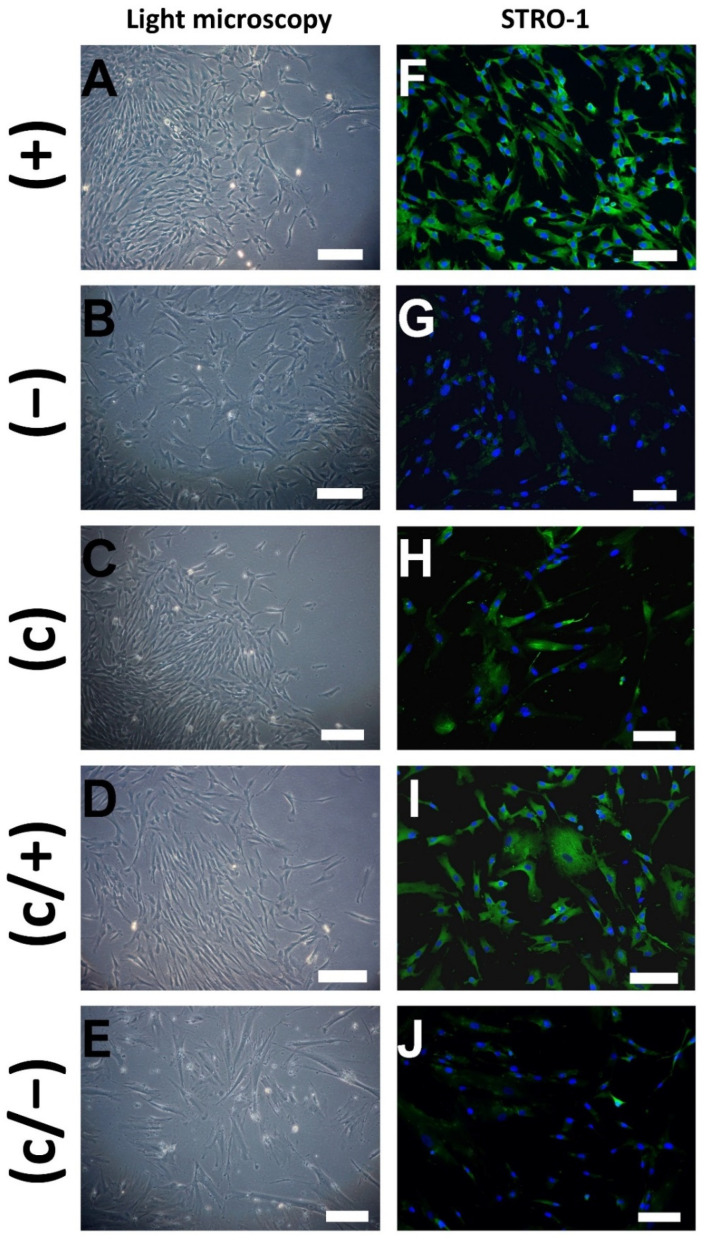
Representative light microscopic (**A**–**E**) and indirect immunofluorescence (IIF) (**F**–**J**) images of different dental pulp cell isolates: (+) MACS-enriched STRO-1+ cells; (−) STRO-1− cells; (c) colony-derived cells; (c/+) colony-derived, MACS-enriched STRO-1+ cells; (c/−) colony-derived STRO-1− cells. (**A**) Small (+) cells grow in colony-like patterns and exhibit a mainly triangular morphology. (**B**) Similar sized (−) cells were equally distributed in the culture dish and are more flattened. (**C**) Colonies emerged by limiting dilution, giving rise to spindle-shaped (c) cells. (**D**) Elongated colony-derived and MACS-enriched (c/+) cells grew in colonies. **(E**) Large-sized (c/−) cells produced extracellular vesicles. (**F**) (+) cells stained positive for STRO-1 upon immunodecoration with fluorophore-labeled antibodies, while positive signals were hardly visible in (−) cells (**G**). (**H**) Colony-derived (c) cells partly stained positive for STRO-1. (**I**) Additional magnetic anti-STRO-1 enrichment yielded (c/+) cells with high STRO-1 abundance. (**J**) Only weak signals were detected in the STRO-1-negative fraction. Green: Fluorescein-labeled STRO-1, blue: cell nuclei (DAPI-staining), scale bar (**A**–**J**): 100 µm.

Following magnetic enrichment, small sized (+) cells grew in colony-like patterns. They were triangular or rectangular in shape, revealing a clear appearance of cell borders in both the longitudinal and the transverse direction, while largely lacking cell extensions and branches (Figure 2A). STRO-1-depleted (−) cells were similar in size but exhibited a more flattened/widespread shape and were equally distributed in the culture dish (Figure 2B). Limiting dilution technique yielded colonies of small, but spindle-like cells, giving rise to (c) cells (Figure 2C). In the combination group, (c/+) cells retained their capacity to form colonies, but exhibited more elongated spindle-like cell shapes, when compared to (+) cells (Figure 2D). Colony-derived, STRO-1-depleted (c/−) cells did not grow in colony-like patterns but appeared large in size, as well as outspread and branched in shape, while producing numerous extracellular matrix vesicles (Figure 2E), which already suggests they were slow in proliferation.

To assess the purity of the cell fractions, samples from all populations were subjected to STRO-1 immunostaining and indirect immunofluorescence (IIF) microscopy shortly after the sorting procedure. As expected, (+) cells stained abundantly positive for STRO-1 (Figure 2F), while positive signals remained sparse in (−) control cells (Figure 2G). In comparison, not all (c) cells stained positive for STRO-1 signals (Figure 2H). Nevertheless, further magnetic enrichment entailed a notable increase in STRO-1+ cells in the (c/+) fraction, when compared to (c) cells (Figure 2I). Only a few positive STRO-1 signals were detected in colony-derived, STRO-1-depleted (c/−) cells (Figure 2J).

In summary, the purification methods led to three cell fractions, i.e., (+), (c), and (c/+) that (i) exhibit a similar morphology, (ii) highly express STRO-1, and (iii) form colonies in vitro. Both control cell populations, i.e., (−) and (c/−), (i) were not able to form cell colonies in vitro, and (ii) mainly stained negative for STRO-1. This shows that colony selection primarily leads to the (antibody-independent) enrichment of STRO-1+ positive cells and that STRO-1- cells are generally not clonogenic.

### 3.2. Differently Purified Dental-Pulp Cells Discriminatively Express Mesenchymal Epitopes

**Figure 3:**
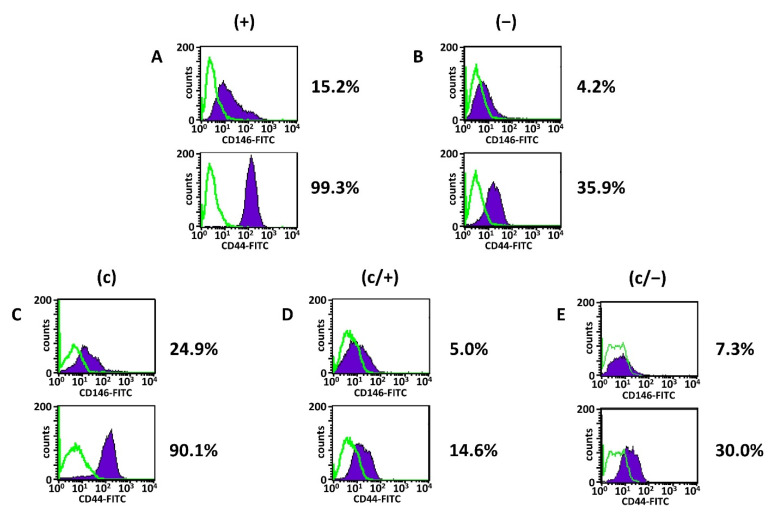
Representative flow cytometric/fluorescence-activated cell sorting (FACS) analyses of different dental pulp cell fractions. (+) MACS-enriched STRO-1+ cells; (−) STRO-1− cells; (c) colony-derived cells; (c/+) colony-derived, MACS-enriched STRO-1+ cells; (c/−) colony-derived STRO-1- cells. Histogram plots of CD44 and CD146 in (**A**) (+) cells, (**B**) (−) cells, (**C**) (c) cells, (**D**) (c/+) cells, and (**E**) (c/−) cells with the corresponding percentages of positively stained cells (mean values and standard deviations are given in Table 1, see below). Details are given in the main text. Green lines: isotype-matched controls, purple areas: positive events; fluorescein isothiocyanate (FITC).

Immunophenotyping helps to characterize the presence of specific epitopes on cells. Thus, the five differently purified dental pulp cell fractions were subjected to FACS-dependent analysis of common surface marker proteins (Figure 3). The selection of the marker proteins is oriented on the panel presented in the “Minimal criteria for defining multipotent mesenchymal stromal cells. The International Society for Cellular Therapy position statement” [74]. Therein, mesenchymal SCs are defined as cells expressing CD73, CD90, and CD105, but lack the expression of CD11b, CD14, CD19, CD34, CD45, and HLA-DR. We thus aimed at finding out to which extent DPSCs fit these criteria.

CD10, also known as Neprilysin, is a metalloproteinase and characterizes several progenitor cell populations, e.g., in the vascular context, where CD10+ cells exhibit a strong clonogenic and osteogenic potential [75]. The aminopeptidase CD13 is found to be expressed by some mesenchymal/mesenchymal-like stem cell populations [76]. CD14 is mainly present on monocytes and macrophages [77], whereas CD19 defines B-lymphocyte populations. HLA-DR, CD34 and CD45 were analyzed as classical hematopoietic cell markers, which are not present on bone marrow-derived mesenchymal SCs (BMMSCs) [78]. Additionally, CD44, CD90, CD105, and c-Kit (CD117) are found on hematopoietic stem cells [79,80], while CD44, CD73, CD90, CD105, CD146, and CD166 characterize human mesenchymal SCs [81,82]. Above, CD44 is a marker protein of osteoblasts [83]. Membrane-bound alkaline phosphatase (ALP) is commonly accepted as a general marker of cells with a high developmental potential [84].

The analysis revealed that irrespective of the isolation method, all dental pulp cell fractions expressed CD13, CD73 and CD90, while the hematopoietic markers CD14, CD19, CD34, CD45 as well as HLA-DR and c-kit were absent (Figure 3A–E, Table 1). (+) cells further expressed high levels of CD105, robust levels of CD44 and CD166 together with intermediate levels of CD10 and CD146 (Figure 3A, Table 1 column 2). The presence of membrane-bound ALP was very low in (+) cells (Figure 3A, Table 1 column 2).

In comparison with (+) cells, the degree of CD10-positive cells was elevated in (−) cells, while the CD44 and CD146 expression were reduced (Figure 3B, Table 1 column 3). Of note, the expression level of CD10 in (c) cells was comparable to (+) cells, while the expression of both CD44 and CD146 was decreased compared to (+) cells (Figure 3C, Table 1 column 4). Limiting dilution technique entailed a slight decrease in CD105- and CD166-positive cells (Figure 3C, Table 1 column 4), and this trend was reinforced following additional magnetic sorting (Figure 3D,E, Table 1 columns 5 and 6). Further, magnetic separation of colony-derived cells yielded an overall decrease of CD10, CD44, and CD146 both in (c/+) and (c/−) cells compared to all other fractions (Figure 3D,E, Table 1 columns 5 and 6). Notably, the ranking of CD146 expression was (+) > (c) > (−) > (c/+) > (c/−) cells, and CD44 expression was similarly strongest in (+) and (c) cells.

FACS analysis of STRO-1 was also performed (Supplementary Figure S1). The analysis revealed that there was no clear difference in the percentage of STRO-1 expressing cells between all five populations. However, cells had to be expanded in culture for a longer period (approximately 2–3 weeks) before enough cells were available for the analysis. The loss of surface-marker proteins of stem cells during culture expansion, especially STRO-1, is a known phenomenon and is extensively discussed in the literature [69,85,86]. The early IIF staining results (Figure 2) together with the methodology of separation were therefore regarded as reliable markers of a high STRO-1 positivity for (+) and (c/+) as well as in part for (c) cell populations.

Thus, the combination of STRO-1 and relatively high levels of CD146, CD44 discriminates (+) and (c) cells from the other dental pulp cell populations. Additional MACS-assisted STRO-1+ enrichment of colony-derived cells does not lead to a useful differentiation between STRO-1+ and STRO-1− cells.

### 3.3. Analysis of Colony Formation Capacity, Stem Cell Marker Protein Abundance, Metabolic Activity, and Cellular Senescence Reveals Relevant Differences between (+), (c), and (c/+) Cells

FACS analysis revealed a remarkable similarity between (+) and (c) cells and STRO-1 positivity is an established marker for DPSCs, making it very likely that (+), (c), and (c/+) populations contain a considerable amount of DPSCs. Above, (−) and (c/−) fractions were partially lacking the ability to properly differentiate into chondrocytes (see Supplementary Figure S3 and below), osteoprogenitors (see Supplementary Figure S2 and below), or neural-like cells (see Supplementary Figure S4 and below), respectively. Therefore, the further results focus on the three cell populations, which putatively harbor the SCs, i.e., (+), (c), and (c/+).

**Figure 4:**
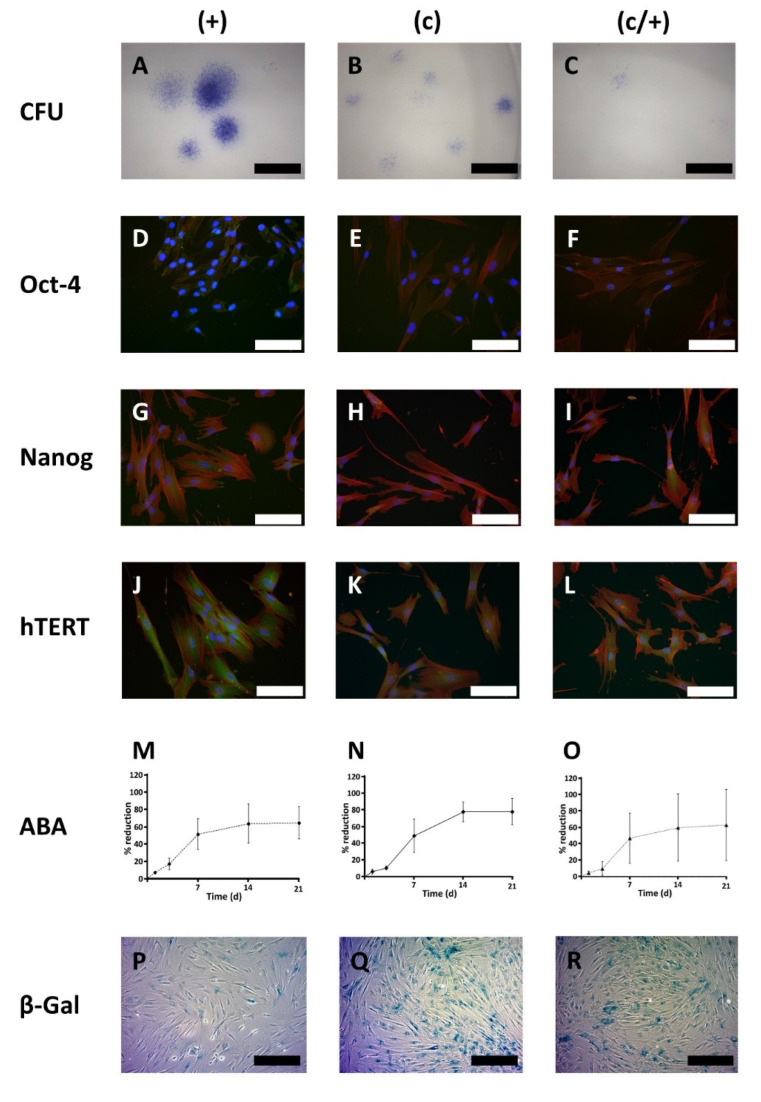
Cell biological characterization of different DPSC populations: (+) MACS-enriched STRO-1+ cells; (c) colony-derived cells; (c/+) colony-derived, MACS-enriched STRO-1+ cells. (**A**–**C**) Light microscopic images of the colony forming units (CFU) assay with Azur II staining. (**A**) (+) cells formed the most colonies with the largest diameter compared to (**B**) (c) and (**C**) (c/+) cells. For the latter cell fraction, only very few small colonies were observed. Scale bars in (**A**–**C**): 600 µm. (**D**–**L**) Immunofluorescence images of the immunostaining of (**D**,**G**,**J**) (+), (**E**,**H**,**K**) (c), and (**F**,**I**,**L**) (c/+) cells for (**D**–**F**) octamer binding transcription factor 4 (Oct-4), (**G**–**I**) Nanog, and (**J**–**L**) human telomerase reverse transcriptase (hTERT). Details are given in the main text. Green: Respective fluorophore-labeled antibody, red: Texas Red-labeled actin fibers, blue: cell nuclei (DAPI-staining), scale bars (**D**–**L**): 100 µm. (**M**–**O**) Metabolic activity of (**M**) (+) [circles connected by a dashed line], (**N**) (c) [rhombs connected by a continuous line], and (**O**) (c/+) [triangles connected by a dotted line] cells as assessed by the Alamar blue^®^ assay (ABA) at day (d) 1, 3, 7, 14, and 21 after cell seeding. (+) initially showed the highest metabolic activity but early entered a plateau phase. Colony-derived DPSCs (c) performed best with regard to the absolute Alamar blue^®^ reduction rate. Colony-derived STRO-1+ cells (c/+) revealed high donor-dependent differences while reaching similar metabolic activity levels as STRO-1+ cells (+). *Y*-axis: Relative Alamar blue^®^ dye reduction; *x*-axis: time scale (days). Error bars represent the standard deviation. (**P**–**R**) Cellular senescence was analyzed with the β-galactosidase (β-Gal) assay and light microscopic images are depicted. Compared to (**P**) (+) cells, (**Q**) (c) and (**R**) (c/+) cells showed a higher proportion of blue positively stained cells. Scale bars (**P**–**R**): 200 µm.

First, the colony formation capacity of the cells was tested in a colony forming units (CFU) assay with Azur II staining (Figure 4A–C). The number of (+) colonies (Figure 4A) exceeded that of (c) (Figure 4B) and (c/+) (Figure 4C) by a proportion of approximately 5.5:2.8:1. Visual assessment also revealed that the diameter of (+) colonies was bigger than that of both other cell populations.

Next, the abundance of classical SC marker proteins, namely Oct-4, Nanog, and human telomerase reverse transcriptase (hTERT) was tested via indirect immunofluorescence (IIF) (Figure 4D–L). In (+) cells, the amount of Oct-4 was highest when compared to the other cell populations, and the protein was predominantly localized in the nucleus rather than in the cytoplasm (Figure 4D). In contrast, (c) and (c/+) cells exhibited a slight cytoplasmic and nuclear positivity for Oct-4 (Figure 4E,F).

Upon analysis of Nanog, all three cell populations, especially (+) and (c/+), stained positive for the protein (Figure 4G–I).

Immunostaining for hTERT proved that the three pulp cell fractions also express this protein, which appeared most abundant in (+) cells.

Next, the ability of the cells to reduce the phenoxazine dye resazurin (Alamar blue^®^) was analyzed after 1, 3, 7, 14, and 21 days (d) (Figure 4M–O). It is an important indicator of cell metabolism and viability and analysis over time is related to cellular proliferation. All dental pulp cell fractions had a curvilinear proliferation pattern with initially low values that significantly increased by time [comparison d 1 vs. d3: (+) *p* < 0.001, (c) *p* < 0.001, (c/+) *p* = 0.025; Figure 4M–O), while (+) cells showed the highest initial increase [(+) vs. (c) *p* = 0.0325, (+) vs. (c/+) *p* = 0.0205, (c) vs. (c/+) not significant). Consequently, intergroup comparison revealed that (+) cells had a significantly higher metabolic activity at d 1 (*p* = 0.012) compared to (c/+) cells (Figure 4M,O). At d 3, (+) cells (Figure 4M) had highest metabolic values compared to both (c) (*p* = 0.066, Figure 4N) and (c/+) cells (*p <* 0.001, Figure 4O). While all cell fractions proliferated rapidly around d 7, (+) cells later entered a proliferative lag phase, as substantiated by confluent cell culture and significantly lower proliferation values at d 14 compared to (c) cells (*p <* 0.001, Figure 4M,N). The absolute metabolic activity levels in (c) cells outrivaled both (+) and (c/+) cells. Of note, (c/+) exhibited an immense donor-related inter-individual differences in cell growth, as indicated by the large error bars (Figure 4O).

Regarding the total time in culture, i.e., the time from cell seeding to reaching confluency, (+) cells grew most rapidly with 20.8 ± 2.1 d, followed by (c) cells with 24.3 ± 1.9 d and by (c/+) cells ranking last with 34.8 ± 10.5 d (data obtained from cell culture; not shown). This observation is both reflected by the respective slopes in the metabolic activity diagrams (Figure 4M–O) and the cellular senescence measured by β-galactosidase activity/X-GAL-staining (Figure 4P–R). It was microscopically obvious that (+) cells exhibited the lowest number of β-galactosidase positive cells (Figure 4P), while in (c) and (c/+) cultures most cells stained positive for X-GAL (Figure 4Q,R, respectively).

In summary, (+) isolates efficiently form colonies and show a high proliferative activity with only few senescent cells. Selection by colony formation, however, promotes cellular senescence and increases the total time in culture. Further, (+) cells most impressively expressed the SC marker proteins Oct4, Nanog, and hTERT, and can, thereby, be expected to contain the most substantial amount of SCs.

### 3.4. All DPSC Fractions Are Highly Inducible to Synthesize a Mineralized Matrix and Exhibit a Similar Chondrogenic and Neurogenic Differentiation Potential

Next, the multilineage potential of the differently isolated DPSCs was checked to analyze whether the cells can form a mineralized matrix (as a proxy for hard tissue formation/dentinogenesis) and differentiate into chondrocyte-like or neuron-like cells in vivo, respectively.

**Figure 5:**
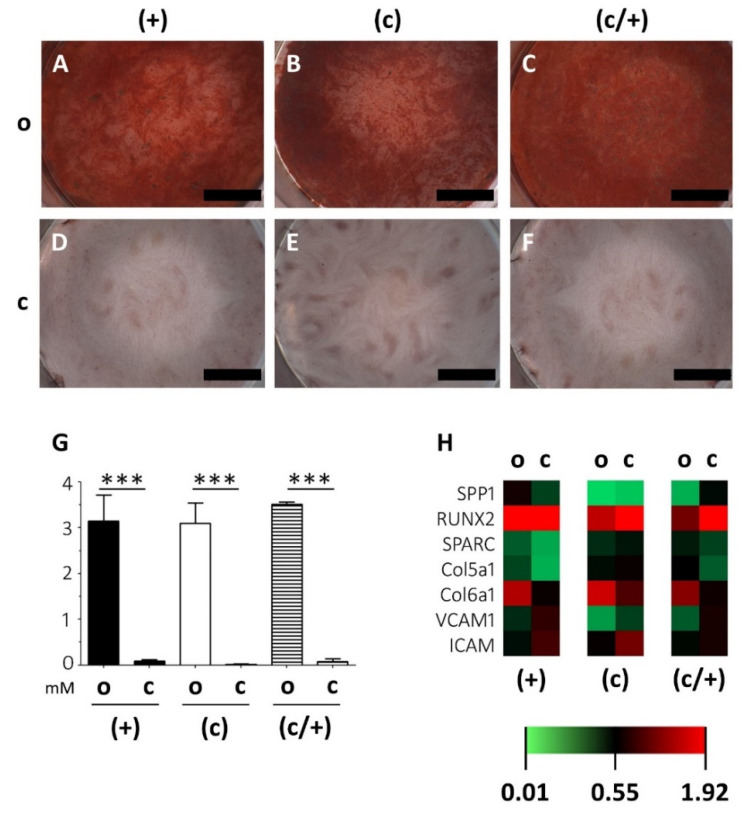
Analysis of the extracellular matrix mineralization potential of osteogenically induced DPSCs fractions. (+) MACS-enriched STRO-1+ cells; (c) colony-derived cells; (c/+) colony-derived, MACS-enriched STRO-1+ cells; o = osteogenically induced; c = non-induced controls. (**A**–**F**) Light microscopic images of the Alizarin red staining of osteogenically induced (**A**) (+), (**B**) (c), and (**C**) (c/+) cells together with the (**D**–**F**) corresponding non-induced controls. Upon induction, all cell fractions can form a mineralized extracellular matrix. Scale bars (**A**–**F**) represent 1000 µm. (**G**) Quantification of the Alizarin red staining via absorbance measurement revealed that matrix mineralization was highly significant in all osteogenically induced DPSC fractions, i.e., (+) [black bars], (c) [white bars], and (c/+) [dashed bars]. *y*-axis: Alizarin red concentration [mM], *x*-axis: osteogenic induction vs. controls. Error bars depict standard deviations. *** *p* < 0.001. (**H**) Gene expression analysis via quantitative PCR showed similar gene expression patterns in all induced DPSC populations with significantly different results only for *COL5A1* and *COL6A1* in (c/+) cells (details are given in the main text). The heatmap depicts the relative fold change in gene expression compared to householding genes (details are given in the Section 2). *SPP1* = Osteopontin, *RUNX2* = Runt-Related Transcription Factor 2, *SPARC* = Secreted Protein Acidic And Cysteine Rich, *COL5A1* = Collagen 5a1, *COLA6A1* = Collagen 6a1, *VCAM1* = Vascular Cell Adhesion Molecular, *ICAM* = Intercellular Cell Adhesion Molecule.

To this end, (+), (c), and (c/+) cells were incubated in osteogenic induction medium for 21 d and the mineralization was assessed and quantified via Alizarin red staining (Figure 5). Control cells from all three populations were accordingly cultured in non-inductive stem cell medium. Strong extracellular matrix mineralization was detected in all DPSC populations (Figure 5A–C) when compared to matched controls (Figure 5D–F). This qualitative, light microscopic observation was confirmed by Alizarin red quantification, which revealed that osteogenic induction entailed a highly significant matrix mineralization in (+), (c), and (c/+) cells (each *p <* 0.001 vs. control, Figure 5G). Regarding the intergroup comparison, the cells from all fractions cultured in osteogenic induction medium reached similar levels of matrix mineralization, with no purification method yielding more intense mineralization than the others. The corresponding results for (−) and (c/−) cells are depicted in Supplementary Figure S2. When compared to the other groups, (−) cells exhibited a reduced matrix mineralization capability.

These findings were substantiated by a very similar transcription pattern of osteogenesis-related genes (Figure 5H; the corresponding expression patterns of (−) and (c/−) cells are depicted in Supplementary Figure S2F). Osteopontin (*SPP1*), Runt-Related Transcription Factor 2 (*RUNX2*), Secreted Protein Acidic And Cysteine Rich (*SPARC*), Collagen 5a1 (*COL5A1*), Collagen 6a1 (*COLA6A1*), Vascular Cell Adhesion Molecular (*VCAM1*), and Intercellular Cell Adhesion Molecule (*ICAM*) were analyzed via PCR and quantified relative to housekeeping genes (see Section 2). Statistically significant results were only obtained for the soft-tissue markers *COL5A1* and *COL6A1*, which were both more intensely expressed in induced (c/+) cells compared to matched controls (Figure 5H). The intergroup comparison revealed no significant differences in the overall gene expression intensity of induced DPSCs irrespective of their method of purification.

**Figure 6:**
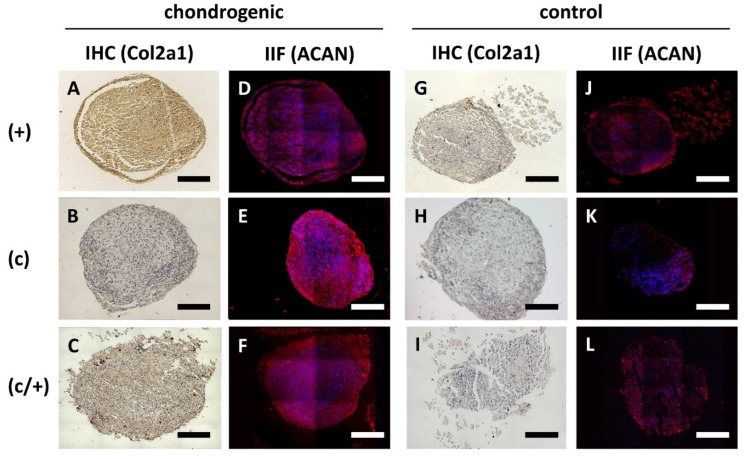
Analysis of chondrogenic induction of different DPSCs populations via immunohistochemistry (IHC) and indirect immunofluorescence (IIF). (+) MACS-enriched STRO-1+ cells; (c) colony-derived cells; (c/+) colony-derived, MACS-enriched STRO-1+ cells. (**A**–**F**) Chondrogenic induction of (**A**,**D**) (+), (**B**,**E**) (c), and (**C**,**F**) (c/+) cells was assessed via (**A**–**C**) immunohistochemical staining of Collagen 2a1 (COL2A1) and (**D**–**F**) immunodecoration of Aggrecan (ACAN). The corresponding controls are represented in (**G**–**L**). All induced DPSC population could be successfully transformed into chondrocytes. The corresponding phenotype is best recognized in (**A**,**C**,**E**). Details are given in the main text. Scale bars (**A**–**L**) represent 100 µm. brown (**A**–**C**,**G**–**I**) = diaminobenzidine staining of COL2A1, red (**D**–**F**,**J**–**L**) = ACAN staining, blue (**D**–**F**,**J**–**L**) = nuclear DAPI staining.

Subsequently, (+), (c), and (c/+) cells were induced to form chondrocytes (Figure 6). Following 21 d of micromass culture in chondrogenic differentiation medium, both (+) and (c/+) cells showed intense signals of immunohistochemical COL2A1 staining (Figure 6A and C). Interestingly, all three cell fractions could synthesize substantial amounts of Aggrecan (ACAN) (Figure 6D–F) with an especially bright fluorescence in (c) cells (Figure 6E). The corresponding non-induced control cells are depicted in Figure 6G–L. Of interest, (c/−) cells barely expressed COL2A1 upon the same treatment, while (−) cells stained positive for this ECM protein (Supplementary Figure S3).

**Figure 7:**
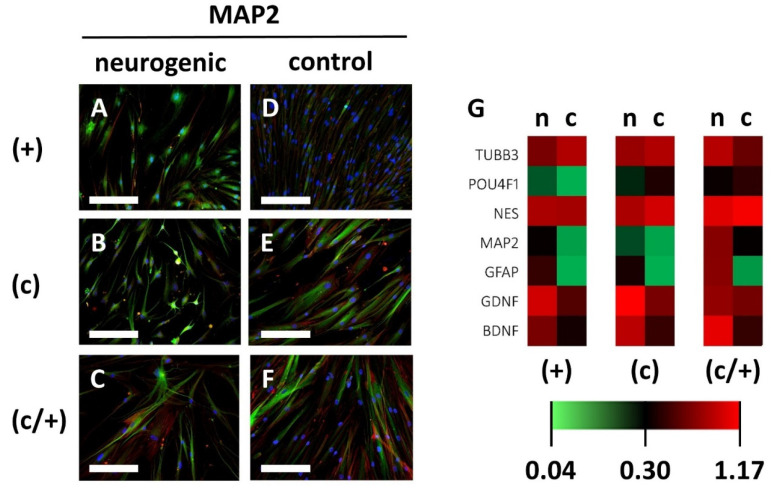
Analysis of neurogenic induction of different DPSCs populations via indirect immunofluorescence and qPCR. (+) MACS-enriched STRO-1+ cells; (c) colony-derived cells; (c/+) colony-derived, MACS-enriched STRO-1+ cells; n = neurogenically induced; c = non-induced controls. (**A**–**F**) Indirect immunofluorescence images of the neuronal marker protein Microtubule-associated Protein 2 (MAP2). Neurogenically induced (**A**) (+), (**B**) (c), and (**C**) (c/+) DPCSs exhibited neuron-like morphologies when compared to the corresponding controls (**D**–**F**). Green: immunodetection of MAP2, red: Texas Red-labelled phalloidin, blue: nuclear DAPI staining. Scale bars (**A**–**F**) represent 100 µm. (**G**) Similar expression patterns were detected for neurogenic genes, with only the *BDNF* gene being significantly upregulated in (+) and (c) cells when compared to matched controls. The heatmap depicts the relative fold change in gene expression compared to householding genes (details are given in the Section 2). *TUBB3* = Tubulin Beta 3 class III, *POU4F1* = POU Domain, Class 4, Transcription Factor 1, *NES* = Nestin, *MAP2* = Microtubule-associated protein 2, *GFAP* = Glial Fibrillary Acidic Protein, *GDNF* = Glial Cell Line-Derived Neurotrophic Factor, *BDNF* = Brain-Derived Neurotrophic Factor.

With regard to neurogenic differentiation, induction of (+), (c), and (c/+) cells for 21 d led to changes in cellular morphology (Figure 7). The induced cells of all three DPSC populations exhibited round and branched cell shapes, which are typical of neurons (Figure 7A–C). Contrary to that, control cells were elongated and spindle-shaped (Figure 7D–F). Upon immunofluorescence imaging, the abundance of the mainly neuronally expressed protein Microtubule-associated protein 2 (MAP2) was higher in induced cells when compared to controls (Figure 7A–F). Morphological changes and adaptation of MAP2 expression were less pronounced in (−) and (c/−) cells (Supplementary Figure S4A–D).

Further analysis of neuronal gene-expression patterns via quantitative PCR on the neuronal marker genes Tubulin Beta 3 class III (*TUBB3*), POU Domain, Class 4, Transcription Factor 1 (*POU4F1*), Nestin (*NES*), Microtubule-associated protein 2 (*MAP2*), Glial Fibrillary Acidic Protein (*GFAP*), Glial Cell Line-Derived Neurotrophic Factor (*GDNF*), and Brain-Derived Neurotrophic Factor (*BDNF*) was performed (Figure 7G, the corresponding gene-expression patterns of (−) an (c/−) cells are depicted in Supplementary Figure S4E). The expression of *MAP2*, *GFAP*, and *GDNF* showed an upward trend in all cell fractions, while *BDNF* was significantly increased in (+) cells (*p =* 0.0474) and (c) cells (*p =* 0.0139) compared to non-induced controls. The increase in *BDNF* expression was non-significant in (c/+) cells. Intergroup analysis showed no significant differences in gene expression intensity between (+), (c), and (c/+) cells following neurogenic induction (Figure 7G).

These data suggest that DPSCs in the (+), (c), and (c/−) fractions globally show a comprehensive multilineage potential, i.e., the ability to synthesize a mineralized matrix, differentiate into chondrocyte-like and neuron-like cells, irrespective of the purification method applied. Contrary to that (−) and (c/−) cells are differentially impaired in their ability to differentiate into the respective cell lineages.

## 4. Discussion

The dental pulp harbors a variety of different cell entities, including DPSCs. They are a promising adult SC population for a broad range of regenerative approaches, being readily available and easy to isolate [87]. Apart from dental pulp and teeth regeneration, DPSCs are intensively investigated in the context of biomechanics [88] and many other biological processes and diseases, including enhanced wound healing or ischemic stroke therapy [88,89,90]. Nonetheless, routine clinical application of these cells for therapeutic procedures is currently limited by the lack of a standardized purification procedure for DPSCs after their initial isolation from the root chamber. Consequently, an enormous amount of biological evidence for their regenerative potential is available in the scientific literature, which has, however, been established by using heterogeneous cell populations [47,91,92]. Thus, the aim of this study was to directly compare, for the first time, different purification methods for DPSCs and to analyze the resulting SC populations in terms of biological, morphological, and molecular properties. To this end, dental-pulp cells were isolated from three different donors and purified either by (i) MACS-assisted STRO-1+ cell enrichment, (ii) limiting dilution and colony formation, or (iii) the sequential application of colony derivation and MACS-assisted STRO-1+ cell enrichment, respectively. Light microscopy and indirect immunofluorescence confirmed the SC-like morphology of (+), (c), and (c/+) cells as well as their expression of STRO-1. Upon FACS analysis, the shared expression of many surface-marker proteins was shown. Efficient colony formation with a low cellular senescence was most pronounced in (+) cells. Alizarin red staining, immunohistochemistry of chondrocytic proteins, and the analysis of neuronal cell markers revealed a similar multilineage differentiation potential for all three DPSC cell fractions. Contrary to that, the STRO-1− fractions, i.e., (−) and (c/−), had a reduced differentiation potential. (−) cells exhibited the lowest amount of Alizarin red staining upon osteogenic induction, chondrogentic differentiation was impaired in the (c/−) fraction and both populations showed little morphological changes when testing the capability for neural differentiation.

MACS-assisted STRO-1+ cell enrichment was chosen as one purification method since it combines certain advantages [93,94]. First, STRO-1 is an established marker for mesenchymal stromal/stem cells in the human body [95]. As DPSCs harbor some molecular features of mesenchymal SCs and STRO-1 positivity has been repeatedly shown for various DPSCs populations, it was regarded as a reliable cell surface marker for DPSC enrichment [71,86,96]. Second, MACS (https://www.miltenyibiotec.com/_Resources/Persistent/b5349effdd595b72195e588aff033be3e24706bd/IM0020021.pdf (accessed on 20 March 2022)) is an easy, reliable, non-toxic, and biodegradable laboratory method with various applications in cell biology and reproducibly enables the sorting of mixed cell populations in a sterile manner, hence, qualifying for putative clinical applications [97,98,99]. The principle can also be scaled-up for the potential use in regenerative medicine and leads to high-yield cell enrichment in comparison with FACS [100]. However, using STRO-1 as a surface marker to select for DPSCs is an a priori approach, which already determines that all DPSCs should express this marker protein or that a cell expressing this protein can be regarded as a SC. This is, however, not necessarily the case since it is, e.g., expressed in endothelial cells [101,102].

To circumvent this problem, a second common approach to purify DPSCs was applied, which relies on a cell biological property of SCs, namely clonogenicity [103,104]. Clonogenicity describes the feature of cells to form cell clones, i.e., colonies of cells originating from a single cell. Since whole tissues and organs are derivates of SCs, clonogenicity can be seen as a basic feature of these types of cells [105,106]. Therefore, limiting dilution was applied to create single cell isolates, which were then checked for their ability to form colonies [107]. Again, this approach is limited by the assumption of DPSCs being clonogenic.

Therefore, colony selection was followed by MACS-dependent STRO-1+ enrichment in a third approach. This sequence led to a double purification of cells, which are colony forming and express STRO-1. Two-step purification protocols for SCs or stable cell lines have also been established in other contexts [108,109]. The sequential purification protocol should clarify the question, if a double selection and thus a DPSC exhibiting both biological properties is favorable in terms of basic SC properties, because the repeated singulation of the cells may prevents them from influencing each other in a paracrine manner [110,111].

STRO-1- cells gained from the MACS eluate as well as colony-derived STRO-1− cells served as control populations for the initial characterization of the dental pulp cell fractions. This is a valid internal control since the cells are derivates of the same dental pulp cell isolate and, to the best of the current scientific knowledge, do not significantly express a core DPSC marker protein, i.e., STRO-1.

Next, the five cell isolates, namely (+), (−), (c), (c/+), and (c/−), were further characterized in terms of their morphology and their molecular properties.

Concerning cellular morphology, (+), (c), and (c/+) fractions were found to exhibit a comparable cell shape with triangular to spindle-like cells. This is similar to the light microscopic DPSC morphology reported by other research groups [112,113,114]. Of note, all three cell populations formed colonies in vitro. While this was expected for (c) and (c/+), where cells were selected for this property, this is noteworthy for (+) cells, which were only selected for their STRO-1 surface antigen. Above, (c/−) cells were not able to form colonies anymore, albeit they were colony-derived initially. This indicates that STRO-1+ dental-pulp cells are clonogenic and that there is a good correlation between cellular morphology, STRO-1+, and clonogenicity here. This finding is substantiated by other SC studies [115]. Exemplarily, Zannettino et al. found that clonogenic bone marrow stromal SCs are part of the STRO-1+ cell fraction within the bone marrow of humans [116].

Immunodecoration of STRO-1 was performed next to confirm the purity of the dental-pulp cell isolates. The experiments were performed shortly after the initial purification since surface antigen loss during the passage of cells is a well-known phenomenon in SC research (see below). As expected, (+) cells exhibited a strong positivity for STRO-1, while it was barely detectable in (−) and (c/−) cells. Interestingly, (c) cells were also largely positive for STRO-1, while (c/+) cells revealed a further enrichment of the SC marker when directly compared to (c). This confirms the methodological suitability of the two-step purification. However, (c/+) cells exhibited inferior SC qualities in nearly all other assays when compared to (+) and (c) cells.

The morphological and STRO-1 staining findings lead to the following preliminary conclusions: (i) STRO-1+ cell enrichment and colony-selection as well as the combination of both techniques lead to morphologically similar cell populations with STRO-1 expression. (ii) STRO-1 expression and clonogenicity are interrelated. (iii) Morphologically different, i.e., spindle-like, dental-pulp cells do barely express STRO-1 and are non-clonogenic. (iv) Removal of STRO-1+ cells from the (c) fraction leads to the loss of clonogenicity. (v) In accordance with these conclusions, (+), (c), and (c/+) cell fractions exhibited a DPSC-like morphology, were clonogenic, and expressed STRO-1, which leads to the assumption that these isolates contain a significant amount of dental-pulp cells with SC properties and might, therefore, be most favorable for applications in regenerative medicine.

The analysis of surface-marker proteins on DPSCs is challenging, because many cellular surface antigens have been investigated in these cells, leading to ambiguous reports in the literature. The review article by Lan et al. offers a comprehensive discussion of CD and other antigens usually found on DPSCs [28]. As described above, there is, however no specific and defining surface antigen that exclusively characterizes DPSCs. Therefore, it is necessary to analyze a whole panel of marker proteins and to find a combination that most accurately defines a dental pulp cell population that exhibits biological properties attributed to SCs.

Generally, DPSCs express no hematopoietic cell markers such as CD14, CD19, CD34, CD45, or HLA-DR [28]. This is, however, also true for (−) and (c/−) cells in our study. Of note, CD34 and c-kit negativity is sometimes questioned for DPSCs [117,118]. Above, all five cell fractions did express CD13, CD73, and CD90. This reflects the expression pattern of human BMMSCs, while precluding all these proteins as specific DPSC markers [74]. STRO-1 was also analyzed via FACS. As can be seen in Supplementary Figure S1, (+), (c), and (c/+) fractions only harbored a small number of cells expressing this antigen. While this seems contradictory at first sight, the phenomenon can be explained by a loss of surface antigens during the passaging of cells. Since FACS analysis requires a substantial number of cells, expansion of the cell fractions for several weeks was a necessary precondition. During early passages, different surface marker proteins change their expression. It is known from the literature that this also applies to STRO-1 in different cell populations. Of interest, the loss of surface antigens can also happen in vivo—either as a physiological adaption to processes such as ageing or during pathological events such as inflammation [69,85,86,119,120]. Moreover, the age of the donors as well as a certain intraindividual variability of surface marker expression contributes to the complexity in analyzing SCs with FACS [121,122,123]. However, the synopsis of the FACS experiments leads to the conclusion that high STRO-1+, CD44+, and CD146+ levels are a common feature of (+) and (c) cells. Both cell fractions are assumed to contain a high number of DPSCs (see above). These findings are underscored by reports in the literature. CD44, a receptor for hyaluronic acid, seems to be an important factor in the odontogenic differentiation of DPSCs [124,125]. High CD44 expression might thus be linked to a strong odontogenic/osteogenic differentiation potential. Positivity for CD146, a cellular adhesion molecule, was also repeatedly described and is linked to dentinogenesis and pulp regeneration but is also a strong marker of cellular stemness [98,126]. Additional STRO-1 enrichment of (c) cells, i.e., the (c/+) fraction, is, however, not useful in discrimination DPSC containing cell populations with the marker panel analyzed in our study.

Until here, the findings in this study strongly indicate that (+), (c), and (c/+) cell populations harbor substantial numbers of DPSCs or might even be designated as proper DPSCs isolates. To further compare these three cell fractions, the subsequent analyses except for the differentiation assays were only performed with these isolates to uncover their respective degree of stemness.

For clinical regenerative approaches, it is essential to yield high cell numbers, because the majority of them are prone to apoptosis or necrosis following transplantation [127]. Hence, an ideal SC population for regenerative treatments is sufficiently pure [128,129], has a high proliferative potential [130,131], shows molecular stemness properties [132,133], has a stable metabolism [134,135], and does not exhibit a relevant cellular senescence [136,137]. These properties were, therefore, analyzed next in (+), (c), and (c/+) cells.

The colony forming capacity is an established proxy for the proliferative potential of SCs [138]. Colony-forming capacity was found to be highest in (+) cells, followed by (c) cells. Upon analysis of classical stemness markers, i.e., Oct-4, Nanog, and hTERT, the cell populations did only reveal slight differences, again with (+) outperforming the others. The simultaneous expression of Oct-4 and Nanog in dental-pulp cells was shown to be related to mesenchymal SC-like properties [139]. This co-expression was strongest in (+) cells and might be linked to the STRO-1+ enrichment (mesenchymal marker protein). Although the functional consequence remains to be determined, it can be speculated that this cell fraction might be most efficient in regenerating mesenchyme-derived cell types.

Metabolic analysis of the cell lines also revealed interesting differences between the population. While (+) cells showed a high initial metabolic rate, these cells entered an early lag phase. This is consistent with a short total culture time (time from defined cell seeding to confluency) and the large colonies in the CFU assay. The property is especially interesting in the context of dental pulp regeneration since the dental pulp has a limited blood supply. On the one hand, cells with a high metabolic rate might, thereby, be susceptible to hypoxic damage, which could potentially limit the application of STRO-1+ enriched cells in dental pulp regeneration [140]. On the other hand, the high proliferative capacity leads to many cells in a short period and therefore increases the possibility of the survival of a sufficient number of cells for regeneration. Surprisingly, first studies have even shown that hypoxia can increase the proliferation, migration, and expression of stemness marker proteins in DPSCs [141,142]. Moreover, it promotes the mineralization of dental-pulp cells [143,144]. (c/+) cells exhibited a strong inter-individual variability in the Alamar blue^®^ assay, which limits its validity (also see discussion on marker expression above). Of note, all three cell populations exhibited a high increase in the metabolic activity around day 7, which is linked to strong proliferation. Thus, this point in time might be relevant for optimizing SC-based dental treatments. SCs could be cultivated ex vivo during the highly proliferative phase and be transplanted after enough cells with a stable metabolism have been established. This, again, supports the notion of (+) cells being beneficial for dental pulp regeneration. Further studies need to shed light into the metabolic fine-tuning of DPSCs, their behavior under hypoxic condition, and the relation of hypoxia and metabolism to stemness and differentiation properties.

Concerning cellular senescence, (c) and (c/+) showed intensive X-GAL staining in the β-galactosidase assay. It is tempting to speculate that colony selection favors cellular senescence in these DPSC fractions since (+) cells barely showed any positively stained cells. To the best of our knowledge, this is the first scientific evidence for this hypothesis. It needs to be further investigated in the future, to ascertain if the mode of purification can directly influence cellular senescence or might even lead to premature SC senescence [145]. It is, however, clear that reducing cellular senescence in DPSCs is an important prerequisite for their reliable application in the clinics. Currently, specific scientific knowledge about the exact processes regulating senescence in DPSCs is scarce [136,146]. In vitro passaging promotes DPSC senescence, reduces the proliferative capacity, and induces a senescence-associated secretory phenotype. Of note, the latter seems to negatively influence neighboring cells. Targeting the 5´adenosine monophosphate-activated protein kinase (AMPK) or preventing the shortening of DPSCs telomers might be possible mechanisms to circumvent cellular senescence and to enable proper expansion and long-term culture of DPSCs, as well as establishing their use in regenerative medicine [68,147,148,149].

Finally, the multilineage potential of all dental pulp populations was tested. As can be seen from the results, the (+), (c), and (c/+) fractions could successfully synthesize a mineralized matrix, chondrocyte-typical proteins as well as neuronal cell markers. These are important characteristics of DPSCs, which are frequently reported in the literature [150,151,152,153,154,155]. The minor differences in the respective expression profiles might be relevant when aiming at the regeneration of a specific cell type. Exemplarily, (c) synthesized less COL2A1 than the other DPSCs fractions and might, therefore, be favorable in odontoblast differentiation, when inhibiting the chondrogenic phenotype is desirable. (−) and (c/−) were not able to reliably differentiate into all three lineages. Morphological changes were barely visible upon neurogenic induction in both cell fractions. Moreover, osteogenic differentiation, as indicated by Alizarin Red staining, was impaired in (−) cells and the chondrocyte-typical expression of COL2A1 was only minimal in (c/−) cells.

The cell biological and molecular characterization of the DPSC fractions showed little but biologically significant differences between (+), (c), and (c/+) cells. (−) and (c/−) fractions did not exhibit true SC properties and therefore served as control cells and most likely represent a mixture of dental pulp resident cells. Taken together, in terms of colony formation capacity, cellular senescence, and the expression of stemness markers, (+) cells seem to outperform the other isolates. It is, however, important to consider that many SC properties in the scientific literature have been investigated with other types of mesenchymal SC and extrapolation of these findings might bias our results towards applying mesenchymal SC characterization criteria on DPSCs, which are, however, derivates of the neural crest.

## 5. Conclusions

The dental pulp is a complex tissue, which harbors a specific stem cell population, called DPSCs. The latter is a promising resource for therapeutic approaches in regenerative dentistry. However, until now, there exists no standardized purification method for DPSCs. Our study, for the first time, compared MACS-assisted STRO-1+ dental pulp cell enrichment with colony-derived DPSCs, and a combination of both methods and identified STRO-1+ as reliable key-player in stemness classification. DPSCs are characterized by a specific morphology, clonogenicity, and STRO-1 positivity. All these criteria were matched by the three populations. However, they differed in terms of CD surface protein expression and cell biological properties. MACS-enriched STRO-1+ cells (+) exhibited the most consistent stemness properties, including a robust metabolic activity, a high proliferative capacity, stemness gene expression, a low cellular senescence, and a reproducible multilineage differentiation potential. Neither colony-derived nor cells purified with the two-step approach could unite all these favorable properties. Based on our results, STRO-1+ cell enrichment appears as an optimized approach for stemness-maintaining DPSC extraction with respect to standardization. The method characterized herein appears not only very promising in basic research, but also encouraging for translational dental-pulp regeneration purposes.

## Figures and Tables

**Table 1:** Summary of the flow cytometric/fluorescence-activated cell sorting (FACS) analyses of different dental pulp cell fractions. (+) MACS-enriched STRO-1+ cells; (−) STRO-1- cells; (c) colony-derived cells; (c/+) colony-derived, MACS-enriched STRO-1+ cells; (c/−) colony-derived STRO-1− cells. Flow cytometric phenotyping revealed that irrespective of the cell purification method, dental-pulp cells strongly expressed CD13, CD73, and CD90, varying amounts of CD10, CD44, CD105, CD146, and CD166, but barely any CD14, CD19, CD34, c-Kit, ALP, or HLA-DR. Mean percentages with the corresponding standard deviations from three independent experiments are presented.

% ± SD	(+)	(−)	(c)	(c/+)	(c/−)
CD10	45.9 ± 40.7	67.3 ± 37.4	47.4 ± 40.4	37.4 ± 39.7	34.4 ± 45.1
CD13	89.9 ± 9.1	87.9 ± 9.9	87.2 ± 14.3	80.5 ± 5.4	79.0 ± 1.6
CD14	0.7 ± 0.8	0.5 ± 0.5	0.6 ± 0.7	1.1± 0.4	0.9 ± 1.2
CD19	0.0 ± 0.0	0.1 ± 0.0	0.0 ± 0.0	0.1 ± 0.1	0.2 ± 0.1
CD34	0.3 ± 0.1	0.1 ± 0.1	0.2 ± 0.3	0.2 ±0.2	0.3 ± 0.1
CD44	63.3 ± 55.0	42.5 ± 46.0	70.5 ± 33.7	26.7 ± 18.8	15.8 ± 12.3
CD45	0.8 ± 1.2	0.2 ± 0.1	0.1 ± 0.1	0.2 ± 0.1	0.5 ± 0.7
CD73	96.0 ± 5.4	96.8 ± 2.9	93.7 ± 4.5	87.7 ± 2.7	88.6 ± 2.6
CD90	96.6 ± 3.0	96.4 ± 4.5	99.3 ± 1.1	99.0 ± 0.5	98.1 ± 1.2
CD105	89.4 ± 13.5	90.8 ± 7.2	86.4 ± 9.2	74.8 ± 16.6	73.7 ± 6.1
CD146	29.6 ± 23.8	11.2 ± 8.8	19.2 ± 14.0	7.8 ± 3.0	4.4 ± 2.7
CD166	67.3 ± 29.0	60.8 ± 27.4	57.7 ± 38.1	49.4 ± 14.8	33.3 ± 12.5
c-Kit	0.1 ± 0.1	0.2 ± 0.1	0.1 ± 0.1	0.1 ± 0.1	0.1 ± 0.1
ALP	0.1 ± 0.1	0.1 ± 0.1	0.3 ± 0.3	0.1 ± 0.0	0.1 ± 0.1
HLA-DR	1.2 ± 1.0	0.9 ± 0.1	0.7 ± 0.3	1.4 ± 0.2	1.4 ± 0.1

## Supplementary Materials

The following supporting information can be downloaded at: https://www.mdpi.com/article/10.3390/cells11203204/s1, Figure S1: Representative flow cytometric/fluorescence-activated cell sorting (FACS) analyses of different dental pulp cell fractions; Figure S2: Analysis of the extracellular matrix mineralization potential of osteogenically induced dental pulp fractions; Figure S3: Analysis of chondrogenic induction of different dental pulp populations via immunohistochemistry (IHC); Figure S4: Analysis of neurogenic induction of different dental populations via indirect immunofluorescence and qPCR.
