# Supplementary material for: Characterization of a Stemness-Optimized Purification Method for Human Dental-Pulp Stem Cells: An Approach to Standardization
Source: Cells. 2022 Oct 12;11(20):3204. doi: 10.3390/cells11203204 (PMC9600643; doi:10.3390/cells11203204)
Supplement: Supplementary file 1 [file cells-11-03204-s001.zip › cells-1918840-supplementary.pdf]

Supplementary Materials

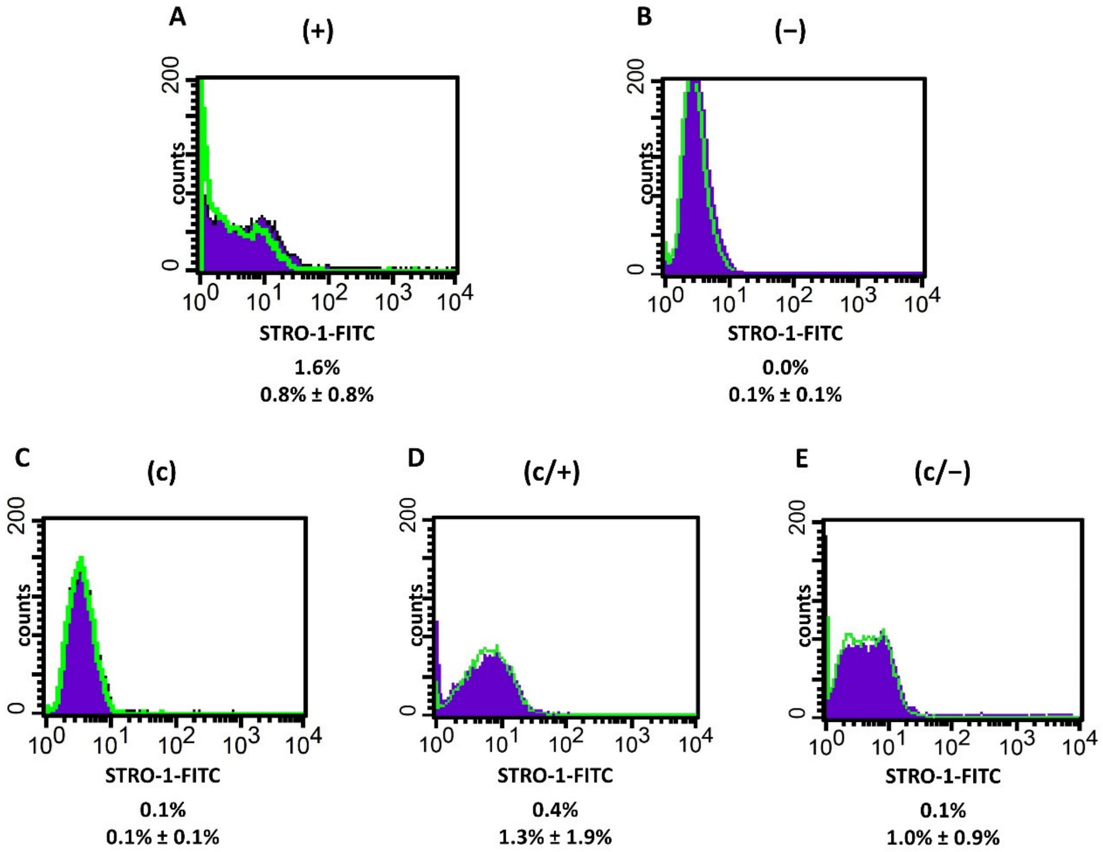

Figure S1. Representative flow cytometric/fluorescence-activated cell sorting (FACS) analyses of different dental pulp cell fractions. (+) MACS-enriched STRO-1+ cells; (–) STRO-1- cells; (c) colony-derived cells; (c/+) colony-derived, MACS-enriched STRO-1+ cells; (c/–) colony-derived STRO-1- cells. Histogram plots depicting STRO-1 expression in (**A**) (+) cells, (**B**) (–) cells, (**C**) (c) cells, (**D**) (c/+) cells, and (**E**) (c/–) cells with the corresponding percentage of positively stained cells in the respective histogram (upper percentage). The mean percentage of positive cells and the corresponding standard deviations from three independent analyses are given in each bottom line. Details are given in the main text (Section 3.2 and Discussion). Green lines: isotype-matched controls, purple areas: positive events; fluorescein isothiocyanate (FITC).

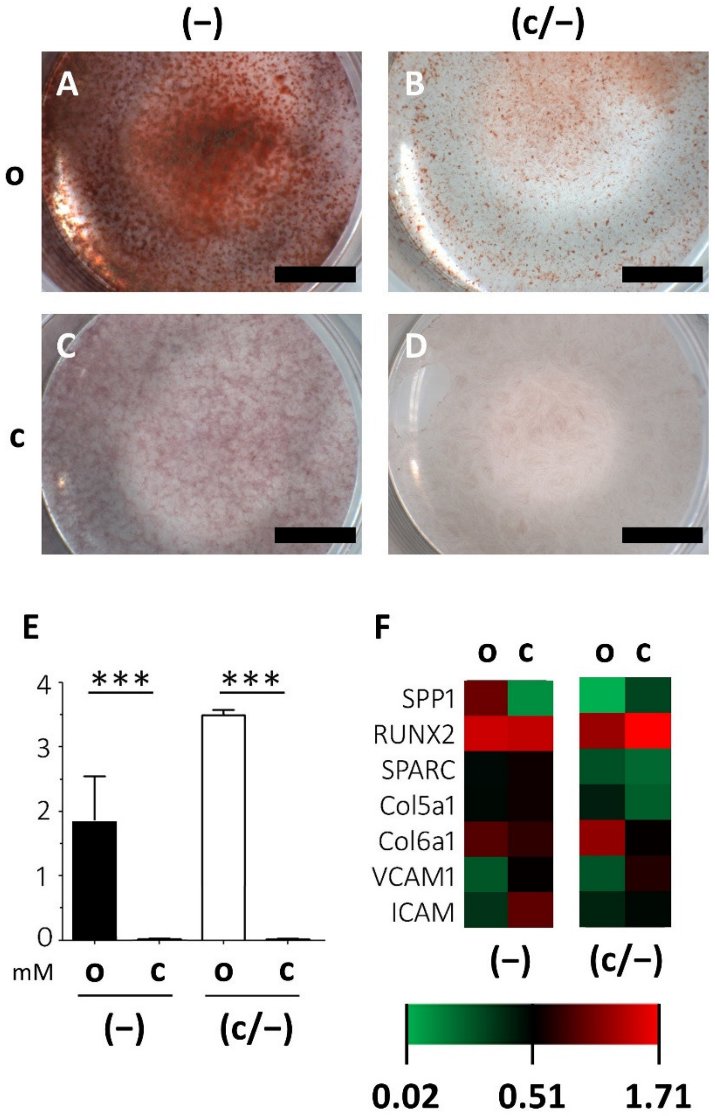

Figure S2. Analysis of the extracellular matrix mineralization potential of osteogenically induced dental pulp fractions. (–) STRO-1- cells; (c/–) colony-derived STRO-1- cells; o = osteogenically induced; c = non-induced controls. (**A**–**D**) Light microscopic images of the Alizarin red staining of osteogenically induced (**A**) (-) and (**B**) (c/−) together with the (**C-D**) corresponding non-induced controls. Note that (**B**) is a representative image from (c/−) cells during osteogenic induction, which does not reflect the quantitatively determined amount of Alizarin Red. Upon induction, (c/−) is more capable of forming a mineralized extracellular matrix, indicating a reduced osteogenic potential of (-) cells. Scale bars (**A**–**D**) represent 1000 µm. (**E**) Quantification of the Alizarin red staining via absorbance measurement revealed that matrix mineralization was highly significant in both osteogenically induced cell fractions, i.e., (–) [black bars] and (c/–) [white bars]. *y*-axis: Alizarin red concentration [mM], *x*-axis: osteogenic induction vs. controls. Error bars depict standard deviations. *** $p < 0.001$. (**F**) Gene expression analysis via quantitative PCR showed differentially regulated gene expression patterns. The heatmap depicts the relative fold change in gene expression compared to householding genes (details are given in the Materials and Methods section). *SPP1* = Osteopontin, *RUNX2* = Runt-Related Transcription Factor 2, *SPARC* = Secreted Protein Acidic And Cysteine Rich, *COL5A1* = Collagen 5a1, *COLA6A1* = Collagen 6a1, *VCAM1* = Vascular Cell Adhesion Molecular, *ICAM* = Intercellular Cell Adhesion Molecule.

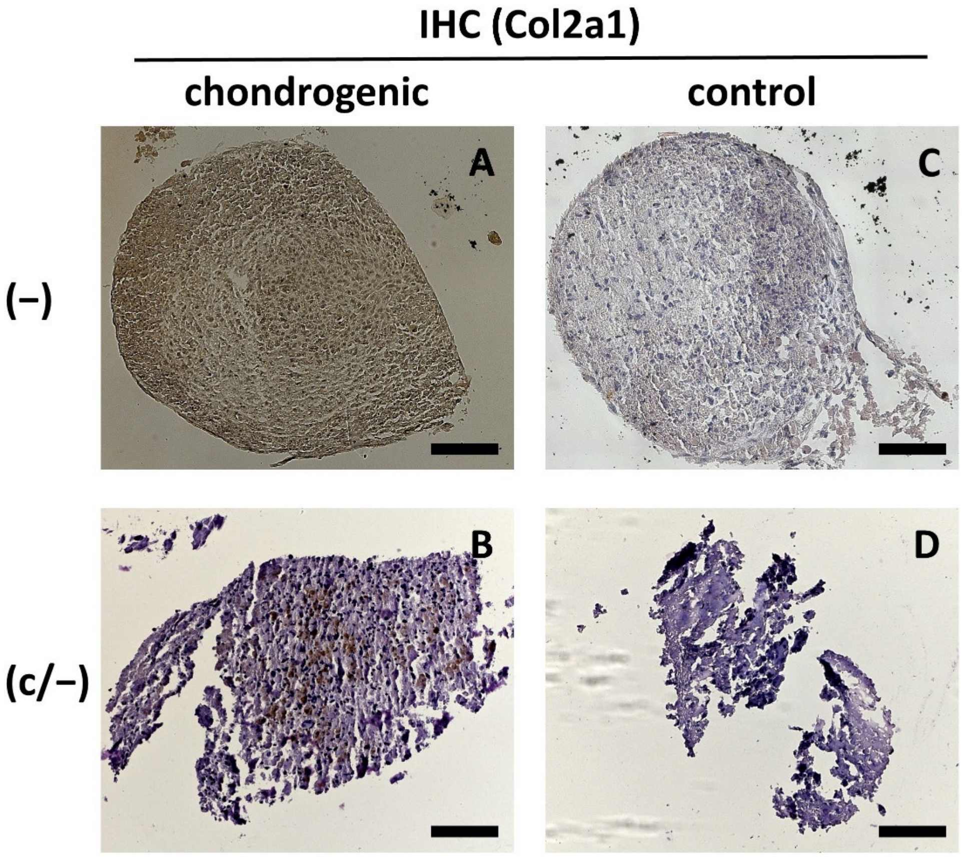

Figure S3. Analysis of chondrogenic induction of different dental pulp populations via immunohistochemistry (IHC). (–) STRO-1- cells; (c/−) colony-derived STRO-1- cells. (**A,B**) Chondrogenic induction of (**A**) (–) and (**B**) (c/−) cells was assessed via (**A,B**) immunohistochemical staining of Collagen 2a1 (COL2A1). The corresponding controls are represented in (**C,D**). Only a few (c/−) cells stained positive for COL2A1, which indicates a reduced chondrogenic differentiation capacity. Scale bars (**A**–**D**) represent 100 μm. brown (**A**–**D**) = diaminobenzidine staining of COL2A1.

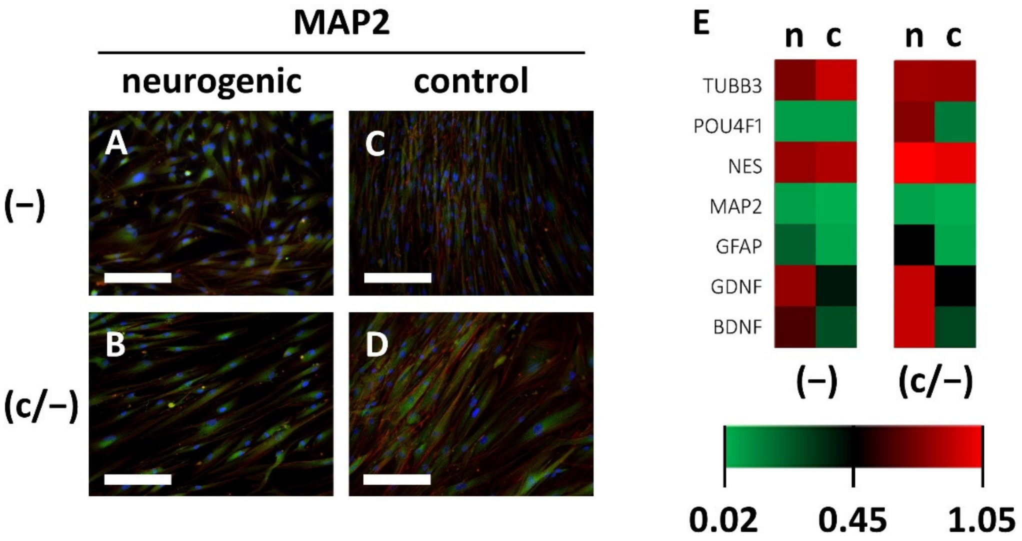

Figure S4. Analysis of neurogenic induction of different dental populations via indirect immunofluorescence and qPCR. (−) STRO-1- cells; (c/−) colony-derived STRO-1- cells; n = neurogenically induced; c = non-induced controls. (**A**–**D**) Indirect immunofluorescence images of the neuronal marker protein Microtubule-associated Protein 2 (MAP2). Neurogenically induced (**A**) (−) and (**B**) (c/−) cells exhibited neuron-like morphologies when compared to the corresponding controls (**C**-**D**). Green: immunodetection of MAP2, red: Texas Red-labelled phalloidin, blue: nuclear DAPI staining. Scale bars (**A**–**D**) represent 100 µm. (**E**) Similar expression patterns were detected for neurogenic genes. The heatmap depicts the relative fold change in gene expression compared to householding genes (details are given in the Materials and Methods section). *TUBB3* = Tubulin Beta 3 class III, *POU4F1* = POU Domain, Class 4, Transcription Factor 1, *NES* = Nestin, *MAP2* = Microtubule-asso-ciated protein 2, *GFAP* = Glial Fibrillary Acidic Protein, *GDNF* = Glial Cell Line-Derived Neu-rotrophic Factor, *BDNF* = Brain-Derived Neurotrophic Factor.
